# The Blood-Fluid

**Published:** 1888-12-15

**Authors:** 


### Physiology and its Uses.

(*Continued from p.* 143.)

### THE BLOOD-FLUID.

Blood, as has been stated, consists of two principal elements —the corpuscles and the fluid. It was explained in a former paper that the blood in natural motion in its vessels could be easily seen in the web of a frog's foot, and that there the corpuscles and the fluid revealed themselves clearly. The liquid portion of the blood is called *plasma* or *liquor sanguinis.* It is a wonderful fluid, both because of what it is and of what it does. When it is completely separated, as by filtration, from the corpuscles, which make it look red, it is seen to be quite colourless and transparent; it looks, in short, like water. But it is water, with such a large number of substances dissolved in it that even to name them all would strike the reader with wonder. It contains albumin, a highly important substance; and fibrin, equally important; fat also, and globulin; with "extractive" matters of various kinds, such as creatin and creatinin, products of worn-out fleshy substance; urea, acids, sugar, colouring matters, odorous matters, salts, etc., etc.

Now let every reader realise to himself that we are speaking of something which is not afar off, something which is not of a speculative kind, which may be either thought about or let alone at pleasure. This substance is a part—a vital, an essential part—of every reader's own body. It is, in fact, the very essence of his health, of his strength, of his pain or of his pleasure, of his life. The blood is the commissariat of the body. It carries to every single organ and tissue the two things without which no organ or tissue can live—air and food. It also carries away from the body certain waste products which are being thrown into it every waking, and probably every sleeping moment of our lives. The very appearance of the blood stream as it flows through the vessels, with its tens of thousands of corpuscles hurrying along in it, unconsciously proclaims the energy and activity of the physiological business which is being conducted by its means. The stream seems as if it were rushing against time; and each corpuscle looks as if it were commissioned with a separate errand of its own, an errand of such urgency that it appears to be striving with all its energy to pass every other corpuscle in the race. The blood current and the active corpuscles know no sleep or waking. Their day is the whole of life; their night is the darkness of death. It is true there are periods of greater and less activity. But from the moment that the newly-formed heart gives its first beat before birth until the day when the last faint stroke proclaims death present, there is no cessation in the wonderful activity of the blood.

The most skilful chemist cannot make blood; it is a cunning mixture which no pharmacy can compound. No physiologist can start off again the vital stream which has once been stopped in death. No anatomist can construct the tubes, the conduits, and the pumping-machine whose motion maintains life, whose rest all the laboratories in the world cannot convert into movement. A few years ago it was thought by some that the physiologist and the chemist were the modern magicians who would put all mystery to flight; whose "sulphur match" of science, as Carlyle called it, was so to illuminate every "cranny and doghole" in nature, that the secrets of life and death were to be secrets no more. Those sulphur matches have merely advanced us a little further into the wide-spreading regions of darkness, and shown us, if possible, more clearly than before, that the night of mystery which envelops mortal man in its gloom is a night which, for mortal man at least, shall never be followed by a noonday. Whatever light death may shed upon the mysteries of human life and destiny, it is certain that upon the greatest of them all physiology and chemistry say little more than they said two thousand years ago. Of the nature of life, of the possibilities of mind and soul, science knows nothing.

But though science can only guess at the underlying mysteries and miracles of life and destiny, the things that are on the surface, and that are of prime importance to health and well-being, are very clearly understood. And herein science shows her kinship with reasonable religion. The mysteries of the Divine nature, of the Trinity, of Immortality, are great, beyond the possibility of sure exploration; but the questions of conscience, duty, charity, and the like—those questions which go to the making or marring of a man's character—these are all as plain and simple as the alphabet to him who brings a single eye and an honest heart to bear upon them. Some persons think such digressions as these out of place in articles whose object is to teach elementary science. But, we ask, does a man want to know science as a parrot knows and tells us that he likes a piece of Stilton—merely because he has learnt to speak it by heart? The intelligent man desires to know the essential facts of all science; the relation between the different sciences; the unity of design that underlies and makes them all one great kosmos. To the man of truly scientific temper, contradictions and accidents have no existence. All science is one, and each understood branch of knowledge is a candle which illuminates and helps to make clear every other branch.

The blood, then, though so full of mystery in itself, yet gives us enough of knowledge to enable us to put it to the highest possible service it is capable of rendering us. It depends largely upon a man's own conduct whether his blood shall be pure or impure, whether it shall carry air and food to his tissues in suitable quantity and quality, or whether it shall carry impure and unwholesome substances, which poison as they go; whether it shall sweep the body clean of waste and injurious matter, or shall leave that matter unremoved, to poison and destroy the organism. The blood is in the fullest sense "the life." Through its chemical laboratory every particle of food, every breath of atmospheric air must pass before it can perform its function in the body. Without it there is no enjoyment of food, no digestion, no assimilation, no repair of the tissues, no growth, no building up. If it be starved, the whole body and the brain are starved; if it be poisoned, body and mind are poisoned too. Just as you cannot have an ocean without water, so you cannot have animal life without blood. Just as a poisoned river surely kills its fish, so does poisoned blood kill, first the small molecules, and finally the whole body. If the blood be in health, the body is in health; if the blood be diseased the body is diseased. If the blood live, the body lives; if the blood die, all the medicine in the world cannot save the body alive. In the whole range of the physician's duties, there is none so important as that of making and preserving for his patients pure blood. In the whole range of a man's own cares there is nothing about which he should be more anxious than pure blood. What pure religion is to the soul, that is pure blood to the body: the one essential and indispensable condition of sound and perfect life.

### TWO HUNDRED YEARS AGO.

The Domestic State Papers for 1667 contain "A true report of the great number of poor children and other poor people maintained in the several hospitals under the pious care of the Lord Mayor, commonalty, and citizens of the City of London," viz.: Christ's Hospital—Children apprenticed last year, 130; buried, 18; remaining in the hospital, 267. St. Bartholomew's—Persons relieved during the year, 1,383; buried from the hospital, 114; remaining under care, 196. St. Thomas's—Persons relieved in the year, 1,241; buried, 144; remaining under care, 255. Bridewell—Returns imperfect, the books being burnt in the late fire, but present numbers, 125. Bethlehem—22 lunatics brought in during the year; 25 cured and discharged; 59 remaining. With recommendations of the said hospitals to charity, the revenue being much lessened in the late fire.

